# 

**Published:** 1889-05

**Authors:** 


					﻿io cts. a copy
MAY, 1389.
$1.00 per year.
HALLS*
\l r
HE Al T H
ESTABLISHED 1854
36*
#>.• 5.
OFFICE: 206 BROADWAY, EVENING POST BUILDING, Room 11. N. Y.
Entered as Second-class Matter at the Post-Office, New York.
				

